# Electrospun Fibrous Scaffolds for Small-Diameter Blood Vessels: A Review

**DOI:** 10.3390/membranes8010015

**Published:** 2018-03-06

**Authors:** Nasser K. Awad, Haitao Niu, Usman Ali, Yosry S. Morsi, Tong Lin

**Affiliations:** 1 Biomechanics and Tissue Engineering Group, Swinburne University of Technology, Hawthorn, VIC 3122, Australia; awadnrc@gmail.com (N.K.A.); ymorsi@swin.edu.au (Y.S.M.); 2 Institute for Frontier Materials, Deakin University, Geelong, VIC 3216, Australia; haitao.niu@deakin.edu.au (H.N.); usman.ali@bzu.edu.pk (U.A.); 3 Electrochemistry and Corrosion Laboratory, National Research Centre, Dokki, Cairo 12422, Egypt; 4 College of Textile Engineering, Bahauddin Zakariya University, Multan 60800, Pakistan

**Keywords:** SDBVs, thrombosis, electrospinning, fibrous scaffold, anti-thrombogenic agents

## Abstract

Small-diameter blood vessels (SDBVs) are still a challenging task to prepare due to the occurrence of thrombosis formation, intimal hyperplasia, and aneurysmal dilation. Electrospinning technique, as a promising tissue engineering approach, can fabricate polymer fibrous scaffolds that satisfy requirements on the construction of extracellular matrix (ECM) of native blood vessel and promote the adhesion, proliferation, and growth of cells. In this review, we summarize the polymers that are deployed for the fabrication of SDBVs and classify them into three categories, synthetic polymers, natural polymers, and hybrid polymers. Furthermore, the biomechanical properties and the biological activities of the electrospun SBVs including anti-thrombogenic ability and cell response are discussed. Polymer blends seem to be a strategic way to fabricate SDBVs because it combines both suitable biomechanical properties coming from synthetic polymers and favorable sites to cell attachment coming from natural polymers.

## 1. Introduction

Atherosclerosis is a cardiovascular disease that is caused by the decreased blood vessel fineness due to the formation of plaques and blockage in the blood flow [1,2]. Angioplasty and surgical bypass are common techniques to treat this disease. In the case of surgical bypass, vascular grafts function to transfer the flow of blood instead of the damaged blood vessel. Vascular grafts are used for the treatment of not only cardiovascular diseases, but also several other cases, such as dialysis, pediatric heart operations, and mesenteric ischemia [3]. However, blood vessel replacement has limited clinical success and it is rather expensive [4]. Although autologous vascular tissues have potential for blood vessel replacement because of its biocompatibility and non-thrombogenic endothelium, harvesting autologous vascular tissues may be difficult for some patients. Therefore, synthetic vascular grafts, such as expanded polytetrafluoroethylene (ePTFE, i.e., Gortex) or poly (ethylene terephthalate) (PET, i.e., Dacron), are developed. Since 1956, these materials have been clinically approved for large diameter blood vessels and the occurrence of thrombosis is negligible owing to the high blood flow and low resistance [5,6]. Although these materials are available and provided clinical efficiency, they have low ratio of patency when employed for small diameter blood vessel (<6 mm). The reported patency rates are 40% after six months and 25% after three years [7]. In addition, these materials are non-degradable, which means that the patient is subject to cardiovascular surgery in the long term due to the inability of tissue growth and remodeling. Therefore, some key criteria must be fulfilled when selecting a polymer to develop synthetic blood vessels, which are processability, mechanical property, morphology and porosity, surface wetting property, degradability, and biocompatibility.

Tissue engineering is an emerging strategy to fabricate artificial vascular grafts (VGs). It aims to the growth of extracellular matrix (ECM) through seeding cells on synthetic scaffolds. Various strategies have been devoted to prepare scaffolds such as self-assembly, drawing, template synthesis, phase separation, wet spinning, electrospinning [8,9], and their combination [10,11]. Scaffolds prepared by fibrous materials are highly desirable, especially from nanofibers, because it has been shown that the natural ECM of the tissues comprises three-dimensional (3D)-dimentioal fibrous structure with fiber size in the range of 50–500 nm [12,13].

Most of nanofiber scaffolds are prepared by electrospinning. Electrospinning enables the fabrication of fibrous scaffolds with fiber diameter ranging from nanometers to micrometers, which have physical properties close to that of natural ECM [12,14]. For instance, the electrospun fibrous scaffolds have high porosity, pore-interconnectivity, and large surface areas, providing suitable surface sites to the cells to adhere, proliferate, and grow [14,15].

This review summarizes the progress in using electrospun nanofibers to develop small blood vessels, i.e., those with a diameter below 6 mm. The biocompatibility studies both in vitro and in vivo are described. State-of-the-art works on functionalization of nanofiber tissue scaffolds for the purpose of promoting biocompatibility or decreasing blood clotting are discussed.

## 2. Native Blood Vessels

Natural blood vessels are classified into three types, namely, arteries, veins, and capillaries. Arteries transfer the blood away from the heart and veins supply the blood back to the heart. Arteriole is the name of small diameter artery and venule is the name of small diameter vein. Capillaries join arteries and arterioles with vein and venules, and they also transfer gases and nutrients from blood to tissues and vice versa.

**Figure 1:**
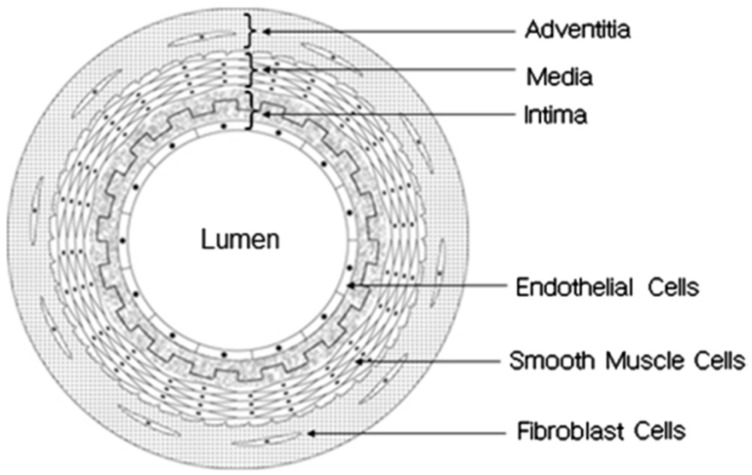
Schematic illustration of blood vessel structure (reprinted from Ref. [16]).

The natural blood vessels have unique composition. The vessel walls comprise of three layers: intima (inner layer), media (middle layer), and adventitia (outer layer), as illustrated in Figure 1 [16]. Intima layer is a monolayer of endothelial cells. Media layer consists of smooth muscle cells (SMCs). Adventitia layer comprises collagenous extracellular matrix (ECM) that carries fibroblast and perivascular nerve cells. Intima, media, and adventitia layers are separated from each other by lamina layer containing elastin. Collagenous adventitia adds rigidity to the blood vessel walls, while lamina provides them with elasticity [16]. Endothelial cell (EC) layer is located at the inner wall of blood vessel, prohibiting the accumulation of blood and adjusting the number of smooth muscle cells (SMCs) in the media layer. Blood vessels dilate and contract in response to a signal from ECs or cytokines [1].

Table 1 lists the mechanical properties of some native blood vessels [17,18,19,20,21]. Confusingly, there is a contradiction between the data outlined for the same tissues due to applications of different evaluation methods. For example, different values for the elastic modulus were reported for saphenous vein in both the circumferential [17,18,19] and longitudinal directions [17,18].

Ideally, synthetic blood vessels should mimic the native blood vessels in both structure and functions. They should be both biocompatible and bioactive, meanwhile, have acceptable mechanical properties.

## 3. Electrospinning Technique

**Figure 2:**
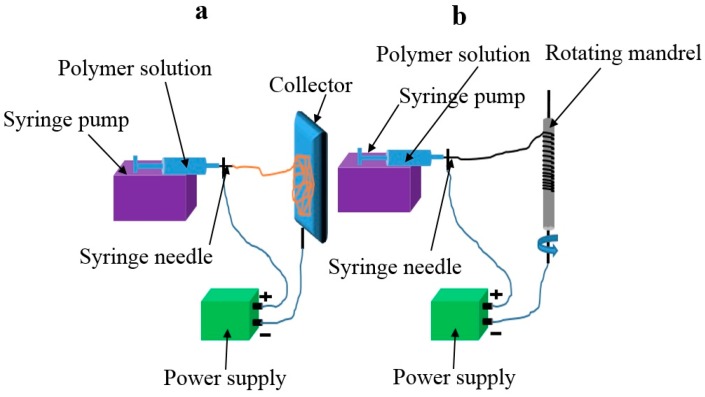
Electrospinning setups: (**a**) grounded flat collector is used to collect fibers; (**b**) tubular rotating mandrel is used to collect fibers to shape blood vessels.

Electrospinning shows great potential in the fabrication of fibrous scaffolds for developing blood vessels. It can produce a seamless fibrous tube with fiber diameter controllable in either nanoscale or microscale. In technology, electrospinning involves a physical process, in which a viscoelastic solution is stretched into solution jet under a high electrostatic force and solidifies to form thin fibers. Figure 2a schematically illustrates a basic setup for electrospinning, which contains a syringe with a syringe pump, a needle nozzle, a collector, and a high voltage power supply. A polymer solution is normally used as electrospinning material. When the solution is charged by high voltage, a Taylor cone is formed at the tip of nozzle. A fine jet ejects from the tip of Taylor cone when the electric field overcomes the surface tension. The charged jet then has an intensive interaction with the electric field formed between the nozzle and the collector, making it undergo a whipping movement. Solvent evaporation from the jet leads to solidification of the solution jet turning into fibers, which deposit onto the collector in the form of randomly-oriented fibrous mats in most of the cases [13,22,23,24].

The fiber morphology of electrospun fibers can be influenced by electrospinning parameters, e.g., solution viscosity, electrical conductivity, polymer molecular weight, applied voltage, flow rate of polymer solution, spinning distance, and ambient condition, such as humidity and temperature [25,26]. Researches indicate that increasing polymer concentration, polymer molecular weight, or solution viscosity leads to increase in fiber diameter. Increasing the electrical conductivity of polymer solution to certain extent decreases fiber diameter. There is a contradictory effect in case of applied voltage [25]. Some papers indicate that applied voltage has no influence on fiber diameter [27], while other papers report that an increase or decrease in fiber diameter could both happen when increasing the applied voltage during electrospinning [28]. The increase of solution flow rate increases the fiber diameter.

In addition, electrospinning technique can be used to directly fabricate tubular fibrous membrane, useful as blood vessel scaffolds, as shown in Figure 2b. Several electrospinning setups have been reported to fabricate blood vessels [29]. The tube diameter can be controlled by adjusting the diameter of the collector.

## 4. Electrospun Fibrous Small-Diameter Blood Vessels

The fabrication of polymer fibrous scaffolds by electrospinning has been intensively investigated. Various polymers have been used including synthetic polymer, natural polymer, and polymer blends. Synthetic polymers exhibit better mechanical properties than natural polymers. Blending two synthetic polymers or two natural polymers could result in enhanced mechanical properties.

Mechanical properties of artificial blood vessels play a major role when the vessels are attached to the native vessels inside the body. If there is a match in the mechanical properties, the sheer stress, as well as intimal hyperplasia can be avoided. Furthermore, the artificial blood vessels should be durable enough to withstand the frequent blood circulation and the associated pressure.

### 4.1. Synthetic Polymer-Based Electrospun Scaffolds

Various attempts have been sought to fabricate artificial blood vessels using synthetic polymers including polycaprolactone (PCL), poly(l-lactide-*co*-*ε*-caprolactone) (PLCL), poly-lactic acid (PLA), polyurethane (PU), l-lactide-*co*-trimethylene carb, polypropylene (Moplen 462R PP), and polylactide (PLA 4060D) [19,30,31,32,33,34,35,36,37,38,39,40,41,42].

#### 4.1.1. Poly(*ε*-caprolactone)

Polycaprolactone (PCL) based blood vessels with inner diameter of 2 mm and wall thickness of 650 ± 15 µm composed of a mixture of micro and nanofibers was fabricated. The fabricated grafts demonstrated good mechanical properties. The longitudinal stress and strain to rupture were 4.1 ± 0.5 MPa and 1092 ± 28%, respectively. The burst pressure and suture retention strength were measured at 3280 ± 280 mmHg and 936 ± 32 g, respectively. The average water leakage and average blood leakage were evaluated to be 32.1 ± 1.3 mL min^−1^ cm^−2^ and 0.87 ± 0.08 mL min^−1^ cm^−2^ at 120 mmHg, respectively [30].

In another work, Poly(*ε*-caprolactone) micro and nanofibrous scaffolds with average fiber diameters of 500–2500 nm and 1.9 µm were fabricated by electrospinning. The diameters of blood vessels were 2 mm and 4 mm, respectively. The fabricated blood vessels showed promising mechanical properties. Both tensile stress and strain stress of the fabricated vascular grafts (2–7.4 MPa and 200–1200%) were higher than that of native blood vessels (1.4 MPa and 100%). In fact, this higher mechanical property is advantageous since the mechanical characteristics might experience a great decrease in clinical conditions when the scaffold starts to degrade and the new natural tissues start to form [31].

Jin-Jia Hua et al. [32] reported that the higher the rotation speed during electrospinning of poly ([epsilon]-caprolactone) resulted in narrower fiber distribution and the higher the elastic modulus. For instance, fibrous scaffolds fabricated at 250 rpm had elastic modulus up to 10 MPa while those fabricated at 1500 rpm had elastic modulus up to 58 MPa.

Small diameter vascular graft was fabricated by electrospinning of poly(l-lactide-*co*-*ε*-caprolactone) (PLCL). The diameter of the graft (2.3–2.5 mm) and the wall thickness of the graft (50–340 µm) increased with the increase of electrospinning time from 10 min–100 min. The fiber diameter was in the range between 700 nm and 800 nm. It was reported that [34] the thinner wall thickness of the synthetic vascular conduit more complaint the synthetic vascular conduit. For example, the stiffness parameter (β) and diameter compliance (Cd) of the thinnest graft (Inner diameter = 2.3 ± 0.1 mm and wall thickness = 49 ± 5 µm) is 6.8 ± 3.1 and 18.7 ± 11.2%/mmHg × 10^−2^, while it is 76.2 ± 18.0 and 2.0 ± 0.2%/mmHg × 10^−2^ for the thickest graft (Inner diameter = 2.5 ± 0.1 mm and wall thickness = 336 ± 21 µm) [33].

**Figure 3:**
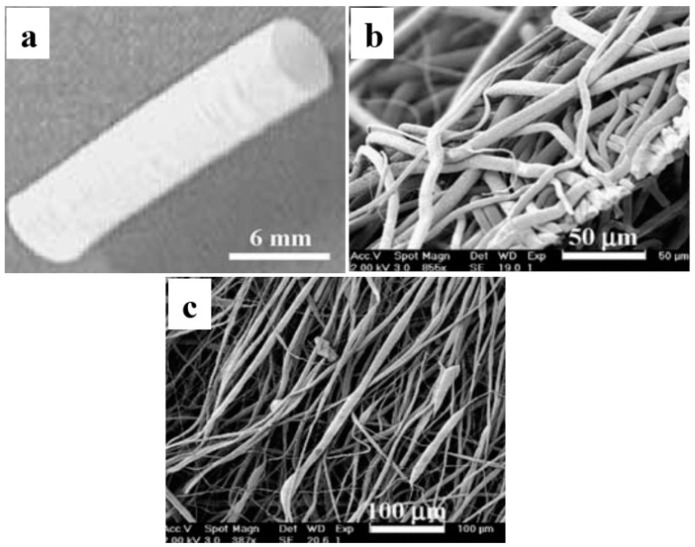
SEM photographs of (**a**) synthetic poly(ε-caprolactone) (PCL)/Poly(l-lactic acid) (PLA) blood vessel of 6 mm diameter, (**b**) the randomly oriented PCL fiber inner layer, (**c**) the aligned PLA fiber outer layer (reprinted from Ref. [34]).

Bilayered tubular electrospun fibrous scaffold comprised pliable polymer Poly-*ε*-caprolactone (PCL) at the inner layer and stiff polymer poly-lactic acid (PLA) at the outer layer was fabricated by layer-by-layer using electrospinning technique, as shown in Figure 3a [34]. Figure 3b illustrates the electrospun PCL scaffold consisting of microfibers and nanofibers with diameters of 1.5 µm to 6 µm and 600 ± 400 nm, respectively, and interconnected pores with 15 µm average pore size. Figure 3c shows the electrospun PLA scaffold consisting of nanofibers with diameters ranging from 800 nm to 3000 nm and interconnected pores with 10 µm average pore size. The total porosity of PCL/PLA scaffold is almost 79 ± 4%. The soft PCL layer mimics the intima layer of natural blood vessel, while the tough PLA layer mimics the adventitia layer of natural blood vessel. The electrospun PCL/PLA fibrous scaffold shows acceptable mechanical properties, with Young’s modulus of 30.9 ± 6.6 MPa almost three times higher than that of PCL scaffold (10.7 ± 0.3 MPa) [34].

Combination of cell matrix engineering with electrospinning technology resulted in fabricating enhanced poly(lactide-*co*-*ε*-caprolactone) (PLCL) nanofiber (1.05 ± 0.23 µm average fiber diameter) based vascular grafts seeded with SMCs in terms of its mechanical characteristics [35]. The cell matrix engineered PLCL vascular grafts demonstrated comparable mechanical properties to native rabbit aorta, as well as high self-sealed property, due to the elasticity of PLCL. For instance, it gave tensile strength values of 1.91 ± 0.56 MPa at a strain of 135% and 3.23 ± 0.57 MPa at a strain of 270% after zero week and four weeks cell culture times, respectively, while the values that were obtained from native rabbit aorta and GORE-TEX were 2.61 ± 0.4 MPa at a strain of 86.7% and 14.03 ± 0.72 MPa at a strain 27.8%. Further, it gave elastic modulus values of 0.85 ± 0.14 MPa and 1.2 ± 0.3 MPa, respectively, after zero week and four weeks cell culture times, while the obtained from native rabbit aorta and GORE-TEX were 0.72 ± 0.1 MPa and 31.61 ± 4.76 MPa, respectively. The burst pressure strength also showed improvement with the increase of cell culture time; it was 604 ± 4 mmHg and 933 ± 22 mmHg, respectively, after zero and four weeks culture time, while it was 1323 ± 383 mmHg and 1647 ± 201 mmHg for GORE-TEX and native rabbit aorta, respectively [35].

Dynamic culture of poly(lactide-*co*-*ε*-caprolactone) synthetic blood vessel using SMCs in bioreactor resulted in an enhancement of the burst strength (1298 ± 156 mmHg) greater than that measured at the static culture (809 ± 44 mmHg) after two weeks [36].

#### 4.1.2. Polyurethane (PU)

PU based vascular graft of 4 mm inner diameter, an average fiber diameter of 732.72 ± 52.22 nm and a porosity of 50–60 showed tensile strength, rupture load, and ultimate elongation values of 5.85 ± 0.62 MPa, 16.5 ± 1.1 N, and 294.5 ± 19.4%, respectively [37]. Thermoplastic polyurethane was modified by pentenoyl chloride of 20% concentration and then cross-linked during electrospinning using UV irradiation. The fabricated blood vessel at diameter of 1.6 mm using the modified electrospun polyurethane fibers showed burst pressure of 550 mmHg and compliance values of 12.1 ± 0.8 and 6.2 ± 0.3%/100 mmHg for uncross-linked and cross-linked fibers, respectively [38].

In another work, thermoplastic polyurethane (TPU) was modified by replacing the aromatic diisocyanate MDI with aliphatic diisocyanate HMDI [39]. Further, the hydrolytic degradability was increased by introducing cleavable chain extenders (CCEs), such as hydroxyethyl lactate (EGLA) and bis (2-hydroxyethyl) terephthalate (BET), instead of the chain extender BDO. TPU containing EGLA exhibited a degradability rate of two times faster than surgical poly (lactic acid) (PLA), while TPU containing BET showed slower degradability rate than PLA. The modified TPU conduits showed tensile stresses less than benchmark Pellethane due to the modification by aliphatic function group [39].

**Figure 4:**
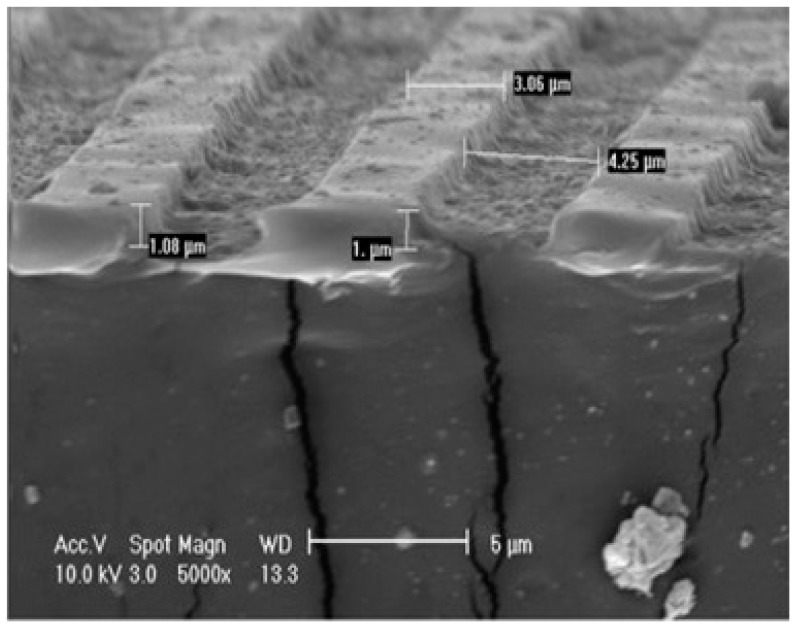
SEM image of polyurethane fibrous scaffold consists of micro-patterned internal layer and electrospun microfiber external layer; the ridge width, channel width and channel depth were 3.6 ± 0.2, 3.9 ± 0.1 and 0.9 ± 0.03 µm, respectively (reprinted from Ref. [40]).

Polyurethane synthetic vascular graft of 4 mm in diameter and 48 mm in length was fabricated by spin casting polyurethane layer first to pattern microgrooves on the lumen area and then electrospinning polyurethane microfibers (average fiber diameter 1.20 ± 0.31 µm) on the outer layer, as shown in Figure 4. The fabricated blood vessel exhibited acceptable mechanical properties with Young’s modulus of 2.00 ± 0.40 MPa and strain stress of 300% [40].

Blood vessels of diameter 4.7 mm and 1.3 mm at lengths of 30 mm and 10 mm, respectively, were fabricated from TIPS/polyurethane ester urea (PEUU) fibrous scaffolds. The inner layer contained porous scaffold of TIPS polymer casted using custom molds that had the same inner diameter of the blood vessels; the outer layer had electrospun nanofibrous scaffold of PEUU, with average nanofiber diameter of 743 ± 201 nm. The pore sizes of TIPS scaffold were 51 ± 3 µm and 123 ± 20 µm, respectively, for 1.3 mm and 4.7 mm blood diameter vessels. The pore size of ES-PEUU nanofibers scaffold was the same in both 1.3 mm and 4.7 mm diameter blood vessels (5.1 ± 3.2 µm). The vessels showed comparable mechanical properties to the native blood vessels. For example, the elastic modulus was 1.4 ± 0.4 MPa, ultimate tensile stress was 8.3 ± 1.7 MPa, and the compliance and suture retention forces were 4.6 ± 0.5 × 10^−4^ mmHg^−1^ and 3.4 ± 0.3 N, respectively [19].

#### 4.1.3. Other Polymers

In addition to the aforementioned two synthetic polymers used as Scaffolds, many other polymers have been reported showing excellent performances. For example, crosslinking l-lactide-*co*-trimethylene carbonate fibrous scaffold by γ-radiation boosted the mechanical properties of the synthetic fibrous scaffolds. The cross-linked scaffold had Young’s modulus matching the native human artery (0.4 to 0.8 MPa) [41].

**Figure 5:**
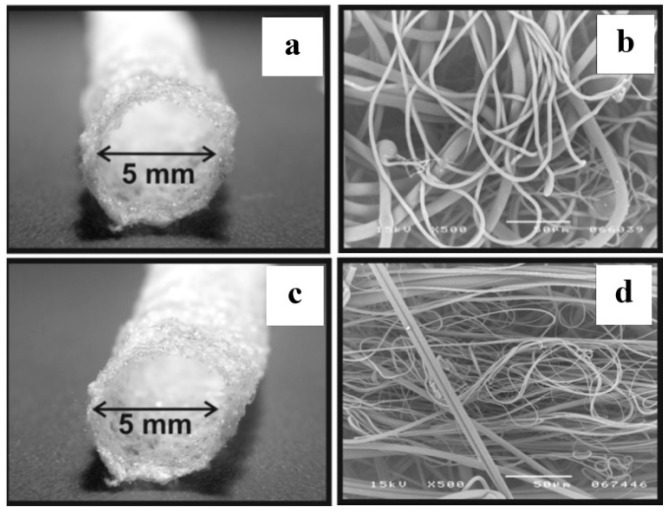
Tubular blood vessels fabricated by melt electrospinning: (**a**,**b**) polypropylene (Moplen 462R PP); (**c**,**d**) polylactide (PLA 4060D) (reprinted from Ref. [42]).

Melt electrospinning technique was employed for the manufacturing of synthetic blood vessel that avoids the drawbacks of using solvents. It was found that the mass flow rate (MFR) had more important influence on the structure of electrospun fibrous scaffold when compared to the other fabrication parameters, such as voltage and distance between the spinneret and the collector. Tubular vascular grafts were fabricated using polypropylene (Moplen 462R PP) and polylactide (PLA 4060D) at the appropriate MFR (25 g/10 min and 2.16 kg at 230 °C), having average fiber diameters of 4.8 µm and 3 µm, respectively, as shown in Figure 5. However, the random nature of the electrospun fibrous scaffolds was obvious, which might affect the response of the cells either positively or negatively [42].

### 4.2. Natural Polymer-Based Electrospun Scaffolds

Natural polymers, such as silk, gelatin, and elastin, have also been employed to fabricate blood vessels however demonstrating low mechanical properties, compared to synthetic polymers [43,44,45,46,47,48,49,50]. Silk-based vascular graft of 5 mm internal diameter and 0.15 mm wall thickness was fabricated. The electrospun scaffold only demonstrated elastic modulus, ultimate tensile stress and burst strength of 2.45 ± 0.47 MPa, 2.42 ± 0.48 MPa, and 811 mmHg, respectively [43].

Juan Zhou et al. [44] optimized conditions for the fabrication of silk fibroin based blood vessels without the utilization of organic solvent that are 18 kV applied voltage, 18 cm collection distance, 37% concentration, and 0.15 mL min^−1^ flow rate. Methanol treatment led to an increment of the tensile strength value from 0.36 MPa to 3.57 MPa. Silk fibroin fibrous scaffold was fabricated by electrospinning, followed by methanol treatment. The treated SF scaffold was more crystalline than untreated SF scaffold, which was confirmed by DSC analysis and ATR-FTIR-ATR. The melting/decomposition temperature and enthalpy measured by DSC analysis shifted to a higher value by increasing methanol treatment time to 15 min, for untreated sample T (°C) and ΔH were 279.70 and 130.13 Jg^−1^, for methanol treated sample T (°C) and ΔH were 287.20 and 138.44 Jg^−1^. FTIR analysis indicated that the peak of β-sheet structure is clearer after long methanol treatment (1699 cm^−1^) [44,45].

Gelatin is considered as collagen-derived product, which constitutes the major ratio of natural blood vessel compositions. Gelatin nanofibrous tubular scaffold was electrospun with an internal diameter of 5 mm and an average fiber diameter of 0.67 µm [46]. Crosslinking of the fabricated scaffolds were achieved by immersing them in 15 mL 25% glutaraldehyde in a Petri dish for three days at room temperature. After that, the cross-linked scaffolds were kept in a fume hood for 3 h to remove glutaraldehyde. Crosslinking is a way for preventing the dissolution of fibrous scaffold when used as vascular graft [47]. The cross-linked scaffolds showed reasonable mechanical properties relative to natural collagen. For instance, the young’s modulus of the cross-linked scaffolds was about 33.8 MPa in the axial direction whilst natural collagen has Young’s modulus of 5–10 MPa [48]. The cross-linked scaffolds further showed an excellent tensile strength of 2.9 MPa in the axial direction when compared to that of 60 KPa measured from human coronary artery. However, the strain to failure of these scaffolds was 11.7% lower than that measured from arteries 35% [49].

The existence of elastin in natural arteries increases the strain to failure and reduces Young’s modulus. Recombinant human tropoelastin (rTE) was utilized for the fabrication of electrospun blood vessels and cross-linked by disuccinimidyl suberate (DSS) [50]. The electrospun rTE fibrous scaffold showed encouraging mechanical characteristics ultimate tensile strength (UTS) of 0.36 ± 0.05 MPa, elastic modulus of 0.91 ± 0.16 MPa, and burst pressure of 485 ± 25 mmHg [50].

### 4.3. Electrospun Scaffolds from Polymer Blends

**Figure 6:**
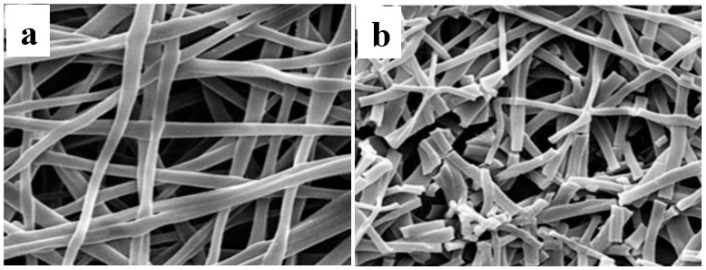
SEM images of in vitro degradation of P (LLACL) fibrous scaffold in PBS at 37 °C after (**a**) 0 time, (**b**) 3 months (reprinted from Ref. [51]).

Polymer blends have been employed as materials to fabricate blood vessels to achieve both good mechanical properties and biocompatibility [51,52,53,54,55,56,57,58,59,60,61,62]. It was found that small diameter blood vessel fabricated from poly(l-lactic acid)-*co*-poly(ε-caprolactone) P (LLACL 70:30) (3 mm internal diameter) had mechanical properties closer to that of native abdominal aorta. For instance, P (LLACL) fibrous scaffold demonstrated tensile strength of 3.9 ± 0.3 MPa in the circumferential direction, while the native abdominal aorta showed tensile strength of 5.29 MPa in the same direction. Figure 6a,b indicated that the fibrous scaffold almost retained its integrity up to 3 month after being immersed in PBS solution at 37 °C [51].

Electrospun PU/PCL blend was used to construct small-diameter blood vessels (3 mm diameter, 0.5–2 µm fiber diameter, 0.5–150 µm pore size). The thus-constructed vessels demonstrated sufficient mechanical properties (tensile strength: 18 MPa, Strain: 375% and pressure strength: 590–600 mmHg) that met the requirement of vascular graft applications [52].

Poly(l-lactic acid) (PLA) and poly(ε-caprolactone) (PCL) (25:75) blend demonstrated better biomechanical properties for cardiovascular graft applications (tensile strength 1.0 ± 0.3 MPa, tensile strain 7.4 ± 2.3%, and suture retention strength 0.454 ± 0.047) than PLA/PCl (75:25) (tensile strength 2.6 ± 0.8 MPa, tensile strain 1.8 ± 1.2%, and suture retention strength 0.062 ± 0.025) [53]. Hybridization of silk fibroin with collagen can result in of small calibre blood vessels (6 mm diameter and 8 mm length) with enhanced the mechanical properties. For instance, silk fibroin-collagen(SF-C) fibrous composite showed burst pressure (894.00 ± 24.9 mmHg) and strain at failure (28.76 ± 1.39%), respectively, while pure silk fibroin fibrous scaffolds (SF) only showed burst pressure (575.67 ± 17.47 mmHg) and strain at failure (27.12 ± 2.63%), respectively [54].

Nanofibrous scaffolds comprised of gelatin/polycaprolactone (PCL) and collagen/poly(l-lactic acid-*co*-*ε*-caprolactone) (PLCL) (average fiber diameter: 386.9 ± 102.5 nm and 301.8 ± 97.3 nm, respectively) were tested in terms of their compatibility as vascular prostheses [55]. Both gelatin/PCL and collagen/PLCL scaffolds demonstrated good wettability (contact angle = 0°). More interestingly, Young’s modulus of collagen/PLCL scaffold increased from 1.77 ± 0.09 MPa to 5.99 ± 0.80 MPa after six weeks transplantation in nude mice as a result of vessel-like tissue formation, whilst Young’s modulus of gelatin/PCL decreased from 1.49 ± 0.06 MPa to 0.75 ± 0.15 MPa after the same period of transplantation [55].

Polydioxanone-elastin (50:50) blend demonstrated similar mechanical properties to that of native femoral artery. PDO (100:0) showed an elastic modulus of 19.98 ± 0.74 MPa, ultimate stress of 5.57 ± 0.7 MPa, and strain at failure values of 206.33 ± 38.96%, while polydioxanone-elastin (50:50) had the values of 9.64 ± 0.66 MPa, 3.25 ± 0.24 MPa, and 64.93 ± 3.97%, respectively, which were approximate to values of Femoral artery (9 to 12 MPa, 1 to 2 MPa, and 63 to 76%) [56].

Collagen-elastin-poly(d,l-lactide-*co*-glycolide) (PLGA) (45%-15%-40%) blend was utilized in fabricating small diameter blood vessel (4.75 mm inner diameter, 477 to 765 nm average fiber diameter, and 0.5 mm wall thickness). The hybrid scaffold demonstrated tensile strength of 0.37 MPa and young’s modulus of 0.85 MPa, respectively [57].

Polylactide-Silk Fibroin-Gelatin Composite based blood vessel (4.5 mm internal diameter, 0.5 mm wall thickness, and 82% porosity) possessed breaking strength, strain, suture retention strength and burst pressure strength values of 2.21 ± 0.18 MPa, 60.58 ± 1.23%, 4.58 ± 0.62 N, and 1596 ± 20 mmHg, respectively [58,59].

### 4.4. Electrospun Layered Fibrous Scaffolds

Polycaprolactone, elastin and collagen have been used to fabricate tri-layered blood vessel resulting in compliance ranging from 0.8 to 2.8%/100 mm Hg and young’s modulus ranging from 2.0 to 11.8 MPa. The compliance and modulus measured for the tri-layered graft equal to that of native artery [60].

Collagen-chitosan-thermoplastic polyurethane (TPU) blends were used to fabricate vascular graft blood vessel (3 mm in diameter). The fibrous scaffolds were electrospun in random and aligned orientations consisting of fibers with diameters of 360 ± 220 nm and 256 ± 145 nm, respectively. Both random fibrous scaffold and aligned fibrous scaffold showed average elongation at break (9.87 ± 1.77% and 58.92 ± 15.46%, respectively) and average tensile strength (9.38 ± 1.04 MPa and 14.93 ± 0.59 MPa, respectively) [61].

Collagen and chitosan improved both the wettability and the biological surface properties of poly(l-lactic acid-*co*-*ε*-caprolactone) P(LLA-CL). Collagen/chitosan/P(LLA-CL) (20:5:75) tubular scaffold of 3 mm diameter, 1.1 ± 0.5 nm pore diameter, 409 ± 120 nm fiber diameter, and 0.33 ± 0.09 mm wall thickness showed ultimate stress (MPa), elongation at break (%), elastic modulus (MPa), burst press (mmHg), compliance (mmHg), and contact angle of 16.9 ± 2.9 MPa, 112 ± 11% mmHg, 10.3 ± 1.1 MPa, >3365 ± 6 mmHg, 0.7 ± 0.4 mmHg and 110.5 ± 0.90, respectively [62].

Table 2 lists the polymers used for the fabrication of small diameter blood vessels and their relative mechanical properties. It is quite clear that the mechanical properties of polymer blends are better than both synthetic polymers and nature polymers.

## 5. Biological Studies of Fibrous Small-Diameter Blood Vessels

Endothelialization of the synthetic blood vessel is a very important issue that affects some factors, including anastomosis (attachment of the artificial blood vessels to the native blood vessels), intimal hyperplasia resulting from the aggregation of particles inside the blood vessels, and thrombosis formation resulting from blood clots.

Various polymers, whether synthetic, natural, or polymer blends, have been subject to in vitro test and in vivo investigating. Natural polymers have better biocompatibility than synthetic polymers, which can promote cell adhesion, proliferation, and growth. The strategy of blending polymers can endow blood vessels with both high mechanical strength from synthetic polymers and the excellent biocompatibility from natural polymers. Blending two synthetic polymers or two natural polymers have also been tried, resulting in enhanced biological properties as well. In this section, several strategies of in vitro tests and in vivo studies will be discussed.

### 5.1. In Vitro Studies

Research on the field of vascular graft fabrication points out that the cell-scaffold material interaction is affected by how stiff the material is. ECs proliferation was found to be declined on stiff gel-based scaffold [69]. Analogously, vascular smooth muscle cell proliferated 20 times higher than that made of polydimethylsiloxane scaffold [70]. In contrary, the proliferation of human dermal fibroblasts only had proportional relation with the stiffness of the matrix [71].

Gaudio et al. showed that Rat cerebral endothelial cells (RCECs) proliferated better on electrospun poly(*ε*-caprolactone) (PCL) scaffolds and electrospun PCL/poly(3-hydroxybutyrate-*co*-3-hydroxyvalerate) (PHBV) composite blends. However, apoptotic cells only appeared significantly on PHBV fibrous scaffold due to the fact that PHBV presented stiffer characteristics [66]. A vascular prosthetic of collagen/chitosan/P(LLA-CL) (20:5:75) promoted ECs cells interaction compared to pure P(LLA-CL) [62]. Encapsulation of vascular endothelial growth factor (VEGF) in the fibrous scaffold of chitosan hydrogel/poly(ethylene glycol)-*b*-poly(l-lactide-*co*-caprolactone) (PELCL) as inner layer and platelet-derived growth factor-bb (PDGF) in the fibrous scaffold of emulsion poly(ethylene glycol)-*b*-poly(l-lactide-*co*-glycolide) (PELGA)/PELCL as outer layer by coaxial electrospinning could potentially control the proliferation of vascular endothelial cells (VECs) and Vascular smooth muscle cells (VSMCs).VECs proliferated faster to cover the lumen of the graft (optical density = 1.4), while VSMCs proliferated slower and adhered to the outer layer of the graft (optical density = 0.4) after three days of culturing [72].

#### 5.1.1. Endothelial Cells (ECs)

Small-diameter grafts (2.4 ± 0.1 mm inner diameter, 12 ± 13 µm wall thickness) made of poly(l-lactide-*co*-*ε*-caprolactone) electrospun nanofibers were subject to endothelialization using human umbilical vein endothelial cells (HUVECs); fiber diameters on the inner and outer surfaces were 799 ± 116 nm and 820 ± 121 nm, respectively. The researchers demonstrated that the gradual increase of shear stress applied on the endothelialized grafts in a custom-designed mock circulatory instrument from 3.2 N/m to 19.6 N/m could reduce the detachment of cells, increase the elongation of cells, and align the cells and actin fibers with the direction of flow. This investigation provided reasonable possibility for the retention enhancement of endothelial cells prior to implantation of the vascular grafts [73].

The adhesion and proliferation of endothelial cells (HUVECs) on PU grafts shows no significant difference that on PTFE grafts in the first three days. From the fourth day, cells had proliferated extensively on PU grafts (absorbency 0.84) than on PU grants (absorbency 0.6) [37].

**Figure 7:**
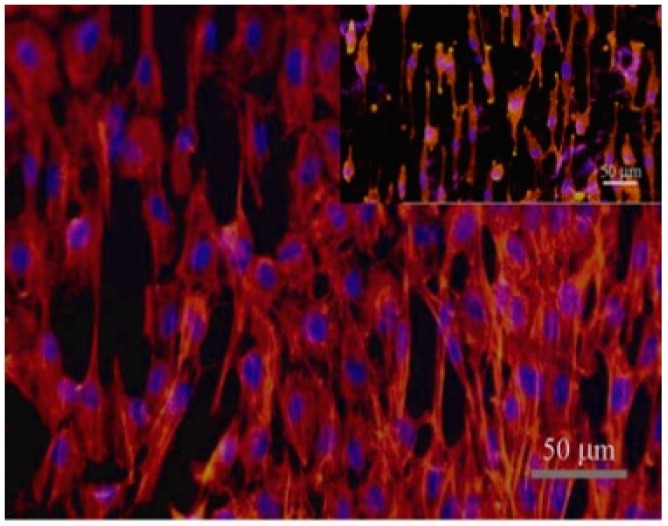
Endothelial cells cultured inside the micro-patterned polyurethane-based synthetic blood vessel after three days (reprinted from Ref. [40]).

The patterned lumen of polyurethane vascular grafts enhanced the full endothelilization of vascular grafts since the cells can migrate through these patterned channels shown in Figure 7 [40]. Aligned nanofibers enhanced BAEC cells response in comparison to random nanofibers when using synthetic blood vessel fabricated from absorbable poly-*ε*-caprolactone (PCL) with 4.5 inner diameter and 400–500 nm average fiber diameter [67].

PU/PCL blend scaffolds resulted in better hydrophilic properties (contact angle: 126°), which supported adhesion, proliferation and growth of cow pulmonary artery endothelial cells when cultured for five days (optical density: 4% versus 1% for 1 day) [52].

Human aortic endothelial cells are cultured on silk fibrous mats. They adhered and proliferated well on the fibrous scaffold. However, human aortic endothelial cells did not migrate under the surface of the fibrous scaffold due to the small size of the pores [43]. Recombinant human tropoelastin (rTE) was used to fabricate blood vessels, which in turn, supported endothelial cell adhesion and growth [50].

Collagen coated P(LLACL) fibrous scaffold by air plasma treatment assisted adhesion, spread, and proliferation of Human coronary artery endothelial cells (HCAECs) after 10 days of culture [51].

Researchers proved that double-layered fibrous scaffolds of poly(*ε*-caprolactone) (PCL) and collagen blend with different fiber diameters (0.27 µm in inner layer and 4.45 µm in outer layer) enabled confluent endothelization on the lumen of the inner layer when tubular vascular graft of 4.75 mm was fabricated [74,75,76].

Tubular scaffold was fabricated using recombinant spider silk protein (pNSR32), polycaprolactone (PCL), and gelatin (Gt) blend. The electrospun pNSR32/PCL/G (5:85:10) tubular scaffold showed porosity, pore size, average fiber diameters and contact angle of 86.2 ± 2.9%, 2423 ± 979, 166 ± 85 nm and 45.7 ± 13.70, respectively. The proliferation of Sprague Dawley Rat Aortic Endothelial Cells (SDRAECs) on pNSR32/PCL/G scaffolds was higher than on pure PCL or even pNSR32/PCL after seven days of culture, providing a higher proliferation index (PI) of 26.8% when compared to that of PCL and pNSR32/PCL (17.8% and 21.5%, respectively) [77]. Similarly, pNSR32/polycaprolactone (PCL)/chitosan (Cs) blend showed PI of 45.79 ± 0.79% and it could also help the seeded SDRAECs cells to release higher concentration of NO within seven days of culture (40 µm/L for PCL/chitosan blend versus 30 µm/L for pure PCL) [78].

Porcine iliac artery endothelial cells (PIECs) were cultured for seven days on both random and aligned collagen-chitosan-thermoplastic polyurethane (TPU) fiber scaffolds, showing almost equal cell viability on both scaffolds for PIECs (absorption index 1.6) [61].

#### 5.1.2. Fibroblast Cells (FBCs)

**Figure 8:**
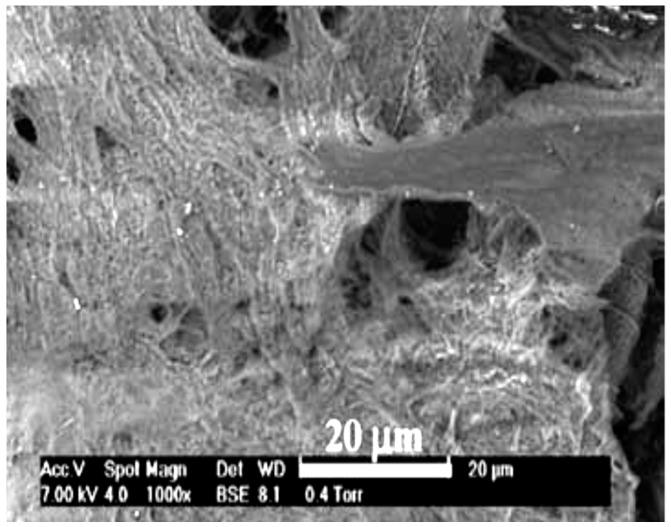
PCL/PLA nanofibrous scaffold cultured in 3T3 mouse fibroblasts cells for 4 weeks (reprinted from Ref. [34]).

Polyurethane grafts provided similar cytotoxicity to the commercial PTFE vascular grafts, and the relative growth factor of mouse fibroblasts (L929) cells was almost 80% in both cases [35]. 3T3 mouse fibroblasts cells adhered well to the surface of PCL/PLA scaffolds made of two layers after four weeks of cell culture shown in Figure 8. Human venous myofibroblasts (HVS) cells were concentrated in the outer layer of PCL rather than in the inner layer of PLA, which was possibly due to the small pore size. However, the cell content was almost 64% comparable to the native porcine pulmonary valve tissue, indicating the progress of tissue growth [34].

Human dermal fibroblasts could penetrate the surface of polydioxanone-elastin, although it was stuck to the surface in case of pure PDO. It was clear that PDO boosted the mechanical properties of the blend, elastin improved the elasticity and cellsinteraction because it can mimics the natural ECM [56]. 3T3 mouse fibroblasts and human umbilical vein endothelial cells experienced good adhesion, spread and proliferation when being seeded on Polylactide-Silk Fibroin-Gelatin fibrous scaffold for 21 days [58,59]. NIH 3T2 fibroblast cells responded better on hybridized silk fibroin-collagen fibrous scaffolds than pure silk fibroin fibrous scaffolds [54].

#### 5.1.3. Smooth Muscle Cells (SMCs)

Poly(lactide-*co*-*ε*-caprolactone) (PLCL) nanofibers based vascular grafts seeded with SMCs showed good biological properties [35], evidenced by the dramatically increased cell viability from 5 × 10^5^ cells to 11 × 10^5^ cells, respectively, with increasing cell culture time from zero week to seven weeks. DNA content evaluation showed enhancement as a result of extending cell culture time from zero week (1.4 ± 0.1 µg/µL) to four weeks (5.6 ± 0.3 µg/µL) [35].

Tubular fibrous scaffolds of poly(lactide-*co*-*ε*-caprolactone) 4 mm internal diameter experienced better SMCs population during the dynamic culture in bioreactor than static culture. Both collagen and DNA contents showed higher expressions in the case of dynamic culture than static culture after two weeks (11.5 μg/mg and 35 μg/mg for collagen, respectively, and 5.7 ± 0.35 mg/µL and 7.5 ± 0.2 mg/µL for DNA, respectively) [36].

A combination of cell sheet technology with electrospinning resulted in harvesting robust confluent cell sheet, which is difficult to obtain by cell sheet technology alone [79]. Firstly, micropatterned polydimethylsiloxane (PDMS) was covered by *N*-isopropylacrylamide (pNIPAm). Secondly, an electrospun polycaprolactone (PCL) scaffold was mounted on the micropatterned PDMS and then cultured by human aortic smooth muscles cells for four days. Eventually, the confluent cell sheet was detached from the PMDS substrate upon cooling to room temperature and rolled over mandrel of 3 mm diameter to form the synthetic blood vessel with contractile SMCs [80].

It has been noted that the expression of cultured SMCs on the electrospun poly(l-lactide) (PLLA) fibrous scaffold covered with polydimethylsiloxane (PDMS) can be either pathogenic synthetic phenotype or contractile phenotype depending on the alignment (random or aligned) of the PLLA scaffold. In the case of pathogenic synthetic phenotype, SMCs were able to swiftly proliferate and migrate producing ECM components, like collagen and elastin. But, in the case of contractile phenotype, SMCs were mature and it would not produce ECM, which was the case of healthy tunica media of natural blood vessel [81,82].

Poly(ester amide) s (PEAs) was used as novel approach for fabricating synthetic blood vessels and PCL was added up to 18–30% to enhance the electrospinnability. PEA-PCL fibrous scaffolds with an average fiber diameter of 0.4 µm enhanced both the proliferation of human coronary artery smooth muscle cells (HCASMCs) and the expression of elastin after seven days culture than PEA discs and even PCL fibrous scaffold of the same fiber diameter [50]. For example, MTT assay revealed that the absorbance of PEA-PCL fibers scaffold (780 nm) was higher than that measured for PCL fibers (450 nm) and PEA films (600 nm). Further, the elastin expression of PEA-PCL fibrous scaffold was 230%, while it was just 50% for PEA films and 100% for PCL fibers [83].

Ovine SMCs seeded on the blended scaffolds of Collagen/elastin/poly(d,l-lactide-*co*-glycolide) (PLGA) (45%/15%/40%), providing mitochondrial 90% metabolic activity after seven days of culture [57]. Human umbilical arterial smooth muscle cells seeded on gelatin/PCL and collagen/PLCL scaffolds for 1 day and showed bipolar spindle shape indicating a contractile phenotype with an optical densities of 0.2 and 0.3, respectively [55].

#### 5.1.4. Mesenchymal Stem Cells (MSCs)

The higher porosity of polycaprolactone (PCL) scaffolds (30 µm and 5–6 µm average fiber diameter) the faster the infiltration of cells and the formation of neoarteries. MSCs cells seeded on high porous PCL resulted in expressing the macrophages in the immunomodulatory and tissue remodelling (M2) phenotype while seeding on less porous PCL led to proinflammatory (M1) phenotype [44]. TIPS/polyurethane ester urea (PEUU) scaffold was tested using adult stem cells, demonstrating cell density of 92 ± 1% [19]. Human mesenchymal stem cells adhered and proliferated well on l-lactide-*co*-trimethylene carbonate fibrous scaffolds crosslinked by γ-radiation [41].

**Figure 9:**
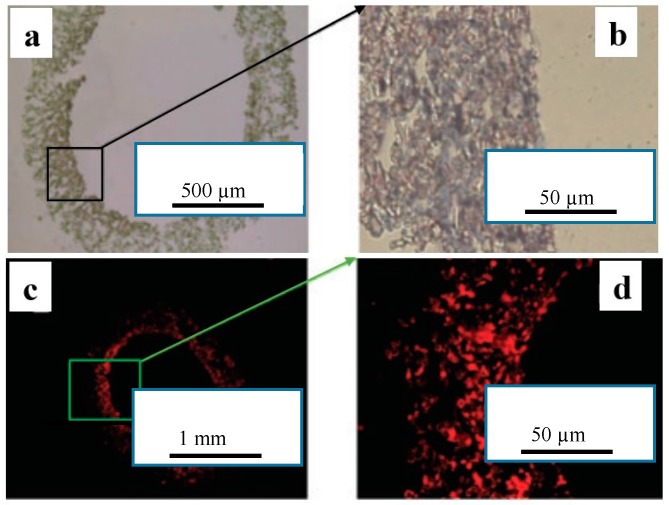
Expression of transgene eNOS protein in both MSCs seeded blood vessel (**a**,**b**) and eNOS-MSCs seeded blood vessel (**c**,**d**) (reprinted from Ref. [84]).

Electrospun poly (propylene carbonate) fibrous scaffold with 5 µm average fiber diameter was used to fabricate vascular grafts (1.5–2 mm in diameter and 0.3–0.4 mm of wall thickness). Bone marrow mesenchymal stem cells (MSCs) were cultured on the vascular grafts for 14 days and they showed acceptable response in terms of adhesion, proliferation, and differentiation. The ability of eNOS modified MSCs seeded blood vessels to produce NO (50 mg/mL) was closer to that detected from fresh rat abdominal artery (68.4 mg/mL) with the same length shown in Figure 9 [84].

### 5.2. In Vivo Studies

**Figure 10:**
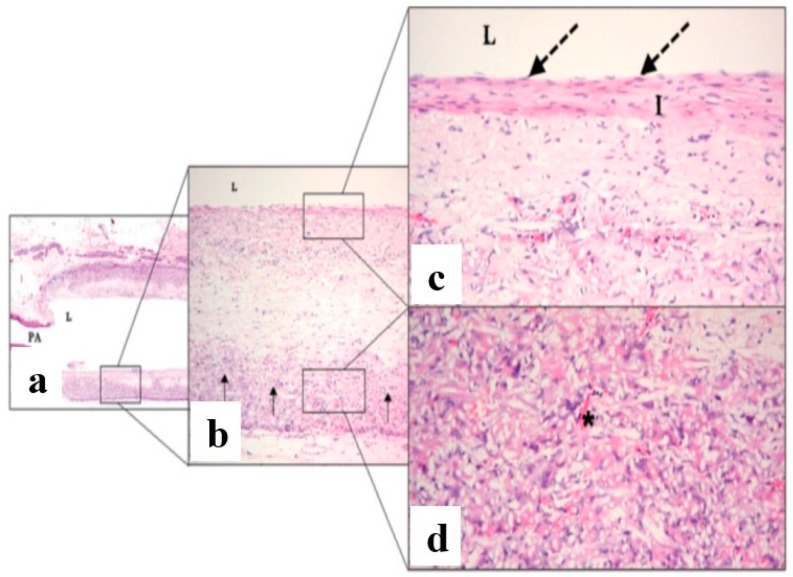
Histological analysis for PCL electrospun blood vessel implanted in an abdominal aortic rat for 12 weeks (**a**) 20 time, (**b**) 100 time, (**c**), and (**d**) 200 time magnification (reprinted from Ref. [31]).

Blood vessel of 2 mm diameter fabricated from Poly(*ε*-caprolactone) (PCL) fibrous scaffold of 1.90 µm average fiber diameter showed better patency rate when compared to expanded polytetrafluoroethylene (ePTFE) grafts when being in vivo investigated in rat for 24 weeks. The growth of endothelial cells and fibroblast cells with extra cellular matrix (ECM) was faster in case of PCL and angiogenesis formation was also observed [63]. The histological analysis of blood vessel made of PCL electrospun fibrous scaffold showed no thrombosis or aneurysm after the implantation of blood vessels in an abdominal aortic rat after 12 weeks. Homogenous infiltration of cells along with the degradation of the scaffold, ECM formation, and full endothelilization were also observed, as shown in Figure 10 [31].

Synthetic vascular grafts (1 mm diameter) that were made of poly(l-lactic acid) microfibers modified by poly(ethylene glycol) and hirudin showed integration and remodelling with host vasculature after being implanted in the carotid artery of female sprague-dawley rats. The elastic modulus increased from 3.5 MPa to 11.1 MPa as a result of the implantation period increasing from one month to six months, due to a significant remodeling of the grafts in vivo [85].

In vivo investigations of 1.3 mm diameter PMA modified conduits made of electrospun biodegradable poly(ester urethane) urea (PEUU) modified with Nonthrombogenic 2-methacryloyloxyethyl phosphorylcholine (MPC) copolymer in abdominal rat for 4, 8, 12, and 24 weeks resulted in patency rate of 92%, which was far higher than that observed for intact conduits (40%). Neotissues comprise of collagen and elastin as well as smooth muscle cells (SMCs) and endothelial cells (ECs) were observed after implantation. Despite the fact that the constructed conduits demonstrated higher compliance (4.5 ± 2.0 × 10^−4^ mmHg^−1^) than native rat aortas (14.2 ± 1.1 × 10^−4^ mmHg^−1^), they reached the native values after four weeks of implantation. Nevertheless, they became stiffer in longer period of implantation [86].

Thermoplastic polyurethane (TPU) conduits demonstrated good biological characteristics when implanted in rat aorta for six months with no thrombin observed [43]. Polyurethane-based tubular vascular grafts were fabricated with 1.5 mm internal diameter, 70 µm wall thickness and 0.88 µm fiber diameter. The fabricated grafts showed 95% patency rate after implantation for seven days, four weeks, three months, and six months in inbred Sprague-Dawley rats. CD34+, myofibroblasts and myocytes cells showed good cell responses in terms of adhesion and proliferation. The ultimate circumferential tensile stress of the grafts was also evaluated to be 26.4 MPa [79,87,88].

PCL-based grafts have not been extensively investigated in vivo for long term [45]. Therefore, Sarra de Valence et al. showed that PCL grafts demonstrated good patency, endothelialization, and no thrombosis was observed up to six months of implantation in abdominal aorta of rat. However, cells regression appeared after 12 and 18 months of implantation due to chondroid metaplasia formation that was responsible for calcification of the grafts.

**Figure 11:**
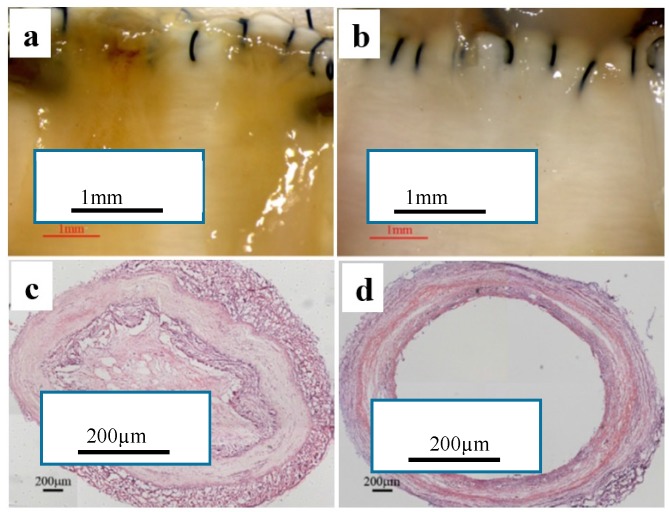
Investigation of explanted (**a**) PCL graft and (**b**) PCL-arginine-glycine-aspartic acid (RGD) graft by stereomicroscope and (**c**,**d**) cross-section staining after four weeks of implantation. Acute thrombosis was observed in the lumen of PCL (reprinted from Ref. [89]).

Small diameter blood vessels of 2.2 mm inner diameter were fabricated by electrospinning polycaprolactone (PCL) comprising of fibrous scaffold with an average fiber diameter of 0.5 to 3 µm and wall thickness of 500 µm. However, PCL surface is hydrophobic and bio-inert. The biomodification of scaffold with arginine-glycine-aspartic acid (RGD)-containing molecule enhanced both patency rate with no thrombosis observed after 4 weeks of implantation and SMCs and ECs infiltration. SMCs covered almost 65.3 ± 7.6% of PCL-RGD surface area after four weeks of implantation, as shown in Figure 11 [89].

In vivo comparison study between polycaprolactone (PCL) and polytetrafluoroethylene (ePTFE) was conducted for 16.5 months. It was evident that PCL characteristics match that of commercial ePTFE in terms of patency rate (100% versus 67%), compliance (8.2 ± 1.0%/100 mmHg versus 5.7 ± 0.7%/100 mmHg), endothelialization (100 ± 0.0% versus 99.6 ± 1.0%), cellular-in-growth (32.1 ± 9.2% versus 10.8 ± 4.0%), and calcification (7.0 ± 5.0% versus 15.8 ± 3.2%). Therefore, this study paves the way for deeper analysis to commercially validate PCL based vascular grafts [90].

Fine mesh polyurethane grafts with low porosity (53%) increased cell adhesion and proliferation at early stages in vivo than coarse mesh polyurethane grafts with high porosity (80%) when implanted in the rat model. However, cell populations were significantly improved by high porosity polyurethane grafts. Fine and coarse mesh grafts both hold the same biomechanical properties before and after transplantation, which were higher than native rat aorta. For instance, fine and coarse mesh grafts demonstrated tensile strength of 20.2 ± 4.6 and 16.3 ± 0.9 MPa, respectively, which were higher than that of native rat aorta 4.4 ± 0.9 MPa [91]. In vivo study of replacing inferior superficial epigastric rabbit veins by P (LLACL) after seven weeks implantation showed that the fabricated scaffold had good patency, with no thrombosis observed [51].

Subcutainous implantation test of the PLA/SF-gelatin fibrous scaffold for three month in sprague-dawley rat showed the absence of macrophages and lymphocytes, formation of vascular network, and shape decrease of the scaffold. This all indicated that the scaffold has good biocompatibility and biodegradability in vivo [58,59].

Hematoxylin and eosin (H & E) staining after subcutaneous implantation of gelatin/PCL and collagen/PLCL scaffolds in nude mice for six weeks showed that gelatin/PCL formed heterogeneous fibers with clear non-degraded scaffold, while collagen/PLCL led to the formation of vessel-like tissues with homogenous surface and bands of collagen [55]. Table 3 lists polymers used for the fabrication of small diameter blood vessels and their biostudies.

## 6. Functionalization of Fibrous Small-Diameter Blood Vessel Scaffolds

Pure fibrous scaffolds without any modifications may encounter thrombosis formation as a result of blood clotting when transplanted in the animal model. Therefore, modification of the fibrous scaffolds by antithrombogenic materials, such as hirudin, lecithin, antithrombogenic polymers, and heparin, or drugs, such as dipyridamole (DPA) and aspirin, could terminate or significantly decrease thrombi formation, enhance endothelialization, and further promote cell proliferation.

Functionalization of fibrous scaffolds could be done either by covalent attachment of the antithrombogenic materials or by mixing antithrombogenic materials or drugs with the electrospinning polymers during the electrospinning process. In this section, several ways for functionalizing fibrous scaffolds are revised to point out the enhancement that occurred for the mechanical properties and biological properties of the fibrous scaffolds after functionalization.

### 6.1. Anti-Thrombogenicity

Craig K. Hashi et al. showed that the modification of poly(l-lactic acid) microfibers with poly (ethylene glycol) and hirudin could reduce platelet aggravation and appear no thrombin activity, due to the existence of hirudin [85].

Lecithin/cholesterol-poly(*ε*-caprolactone) (Chol-PCL) electrospun fibrous scaffolds (average fiber diameter 0.5 to 1 µm) showed better hemcompatibility and cytocompatibility than net Chol-PCL, due to the zwitterionic property of lecithin. The hemolysis ratio (HR), which indicates the extent of broken blood cells at the interface within the scaffold, was much lower in case of lecithin/Chol-PCL (0.5%) than pure Chol-PCL (2.8%). Furthermore, the Lecithin/Chol-PCL conjugate demonstrated biomechanical characteristics, including Tensile strength, Elongation at break (%), and Young’s modulus of 5.22 ± 0.50 MPa, 107.15 ± 10.78%, and 35.92 ± 4.75 MPa, respectively. Bone-marrow mesenchymal stem cells (MSCs) proliferated better on Lecithin/Chol-PCL with optical density of 0.9 nm higher than that on pure Chol-PCL (0.65 nm) when being cultured for seven days [68].

Biodegradable poly (ester urethane) urea (PEUU) was used to fabricate conduits of small diameter (1.3 mm internal diameter) followed by internally immobilization using nonthrombogenic 2-methacryloyloxyethyl phosphorylcholine (MPC) copolymer [86]. Firstly, the surface of PEUU fibrous scaffold was aminated with amine groups using radio frequency glow discharge in ammonia atmosphere. Following this, the amine sites reacted with the carboxyl groups of the PMA polymer through condensation reaction to yield PMA functionalized PEUU fibrous scaffolds. The PMA modified conduits showed reasonable biological activities when exposed to ovine blood since less platelet adhesion observed when compared to untreated conduits [86].

Biodegradable poly(ester urethane) urea (PEUU) and non-thrombogenic bioinspired phospholipid polymer (poly(2-methacryloyloxyethyl phosphorylcholine-*co*-methacryloyloxyethyl butylurethane) (PMBU)) blend was used for the fabrication of small diameter blood vessels (1.3 mm internal diameter, 300 µm wall thickness, and 500 nm average fiber diameter). The PEUU/15% PMBU blend demonstrated Young’s modulus, strain, and compliance of 3 ± 1 MPa, 342 ± 43%, and 4.4 ± 1.1 × 10^−4^ mmHg^−1^, respectively, which were greater than that of pure PEUU (2 ± 1 MPa, 388 ± 58% and 2.9 ± 0.6 × 10^−4^ mmHg^−1^, respectively). The blended scaffold of PEUU/15% PMBU inhibited platelet deposition as well as inhibited rat smooth muscle cells growth in vitro (RSMCs adhesion 70–76% after one day). However, the vivo study of replacing rat abdominal aorta by PEUU/15% PMBU for three months denoted that the fibrous scaffold had patency rate of 67%, which was higher than that of pure PEUU (40%) [63].

Poly-*ε*-caprolactone (PCL) incorporated with peptide cysteine-alanine-glycine (CAG) was utilized to fabricate electrospun small-caliber vascular grafts (SCVGs) of 0.7 mm diameter and 7 mm length. The synthetic grafts were transplanted into the carotid arterial of sprague-Dawley rats for 6 weeks. It was found that CAG containing grafts achieved higher endothelization ratio (97.4 ± 4.6%) than pure PCL-based graft (76.7 ± 5.4%) after six weeks of implantation. On the other hand, α-smooth muscle actin (ASMA) measured for a CAG/PCL graft (0.89 ± 0.06) was significantly less than that of pure PCL graft (1.25 ± 0.22). Therefore, it is speculated that CAG/PCL grafts can enhance endothelilization and inhibit intimal hyperplasia [92].

Combination of fused deposition (FDM) with electrospinning for fabricating vascular conduit resulted in an enhancement of the overall biomechanical and biological characteristics [65]. Poly-l-lactide (PLLA)/heparin (Hep)/poly-*ε*-caprolactone (PCL) based blood vessels (5 mm diameter, 0.3 mm wall thickness, 6 cm length, and 450 ± 150 nm average fiber diameter) showed an ultimate tensile strength of 1.58 ± 0.07 MPa, which was higher than that of electrospun poly-l-lactide (PLLA)/heparin (0.72 ± 0.03 MPa) and human saphenous vein sample (SV) (1.15 ± 0.13 MPa), owing to the existence of PCL coil layer deposited by FDM. Furthermore, live/dead assay (>90% viable cells) and DNA content (4500 ng) showed high viability and proliferation of human adult bone marrow mesenchymal stem cells (hMSCs) cultured on PLLA)/heparin/ PCL for 48 h [65].

Biomimetic small-diameter blood vessels (3 cm in length, 4 mm in inner diameter and 0.25 mm in thickness) were fabricated by electrospinning gelatin-heparin (inner layer) and polyurethane (PU) (outer layer). Both gelatin-heparin inner layer and PU outer layer demonstrated fiber diameter between 108 nm and 174 nm, 587 nm to 1081 nm, respectively, average pore size 1.34 μm, and 1.60 μm, respectively. The thus-fabricated vessels acquired sufficient mechanical characteristics, breaking strength (3.7 ± 0.13 MPa), and elongation at break (110 ± 8%). The release rate of heparin was in the range of between 18.5% (1 day) and 33.0% (14 day). Consequently, it sounded that the PU/Gelatin/Heparin based blood vessels held the structure of native vessels as well as it was hemcompatible as a result of heparin release [93].

Heparin-poly(*ε*-caprolactone) conjugate was employed for the construction of small-diameter blood vessel (Length = 4 cm, Diameter = 2 mm). PCL-heparin conjugate showed hydrophilicity (70°) higher than pure PCL (10°). Since the surface of PCL-heparin conjugate was negatively charged, it suppressed the adsorption of plasma protein like albumin and fibrinogen. Theoretically, the values of albumin and fibrinogen should be 250 and 270 ng cm^−2^ respectively. Experimentally, the values were 500 ± 32 and 560 ± 40 ng cm^−2^ for pure PCL and 330 ± 21 and 340 ± 28 for PCL-heparin conjugate, respectively. ECs cells achieved higher relative growth rate (RGR) in case of culturing on PCL-heparin conjugate (160%) than pure PCL (100%). In vivo investigation was performed in dog’s femoral artery using PCL-heparin graft for four weeks, resulting in potent and compatible graft. One can conclude that the heparin containing graft is opportune to regenerative medicine since it is capable of supressing thrombus formation [94]. Along the same lines, heparin was linked to the surface of poly(l-lactide) (PLLA) by di-amino-poly (ethylene glycol) (PEG), which resulted in a greater patency rate (85.7%) than untreated PLLA (42.9%), and promoted EC and SMC infiltration in the nanofibrous scaffolds [95].

Electrospun heparin/poly(l-lactide-*co*-*ε*-caprolactone) (P(LLA-CL) fibrous scaffold demonstrated higher patency rate of 100% for two weeks implantation in canine model when compared to P(LLA-CL). Furthermore, pre-endothelilized heparin/P(LLA-CL) fibrous scaffold got mechanical properties (tension of 95776 ± 193 g/g and elongation of 8.8 ± 1.7 mm) higher than that of pure P(LLA-CL) and even heparin/P(LLA-CL) [96].

Bionic double layer small-diameter vascular graft (SDVG) of 2.5 mm diameter and 6 cm length was constructed using heparin-conjugated polycaprolactone (hPCL) as inner layer (0.15 µm average fiber diameter) and polyurethane (PU)-collagen blend as outer layer (0.2–1 µm average fiber diameter). The constructed SDVG showed porosity of 45% and burst pressure of 300 kPa. In terms of its biocompatibility, in vitro culturing of L929 fibroblast cells on the inner and outer scaffolds of SDVG separately resulted in cell’s relative growth rates (RGR) of 103.5% and 98.0%, respectively. Moreover, in vivo transplantation of SDVG in beagle dog model for almost eight weeks showed no aneurysmal dilation, extravasation, and stenosis [97].

Tri-layered electrospun small-diameter vascular conduit (1.5 mm diameter and 300 µm wall thickness), was constructed by *co*-electrospinning using poly(*ε*-caprolactone) (PCL) and natural polymer chitosan (CS). The PCL/CS conduit was loaded by heparin, which attached to CS through ionic bond. The internal layer of the conduit had higher concentration of CS (PCL/CS = 5/4 *w*/*w*), which in turn absorbed higher concentration of heparin. Heparin conjugation led to remarkable anticoagulation effect, which was proved by increasing activated partial thromboplastin time (APTT), thromboplastin time (TT) and prothrombin time (PT) (180 s, 150 and 14 s, respectively), as well as decreasing platelets adhesion (PCL/Cs 5/4 *w*/*w*: 100, PCL/Cs 5/4 *w*/*w*: 200). The PCL/Cs tube demonstrated acceptable tensile strength and young’s modulus of 9 MPa and 7.8 MPa, respectively. In vitro test using EC and SMC cells showed that heparin loaded PCL scaffolds promoted the proliferation of EC cells by the secretion and stabilization of VEGF while inhibited moderately the proliferation of SMC cells by the activation of intracellular pathways (O.D of ECs: 0.15 and O.D of SMCs: 0.1 after 1 day). This result met the requirement of blood vessel regeneration because the high proliferation of SMC cells may lead to intimal hyperplasia, especially at the initial stages. Both ex vivo shunt and in vivo implantation in rat abdominal aorta for 1 month confirmed in vitro results demonstrating the absence of any thrombus formation and blood leakage [98].

### 6.2. Drug Loadings

The incorporation of dipyridamole (DPA) into biodegradable elastic polyurethane urea (BPU) fibrous scaffolds during electrospinning for the fabrication of small-diameter blood vessels (1.5 mm diameter, 150 µm wall thickness, 520 ± 100 nm to 650 ± 160 nm average fiber diameter) led to enhancement, both in the biomechanical properties and the biocompatibility. BPU + 10% DPA provided tensile strength and strain value of 7.4 ± 0.1 MP (versus 3.4 ± 0.4 for pure BPU) and 107 ± 20%, respectively. BPU + 10% DPA inhibited platelets adhesion (TAT concentration: 0.6 µg/mL against 1 µg/mL for pure BPU) and SMC cells proliferation, while it enhanced EC cells proliferation after seven days culture [99].

## 7. Conclusions

Synthetic polymer, natural polymer, and hybrid polymer-based scaffolds have been intensely used for the fabrication of small-diameter blood vessels. Electrospinning technique has advantages over the varieties of other techniques that are used for the fabrication of fibrous scaffolds, because it fabricates fibrous scaffolds with average fiber diameters in nano size from 50 to 500 nm. This fiber sizes match that of natural ECM of native blood vessels. Nevertheless, electrospun nanofibers exhibit some limitations, including the use of organic solvents, flat fibrous mat with limited control of pore structure, and relatively low nanofiber mat strength, which need to be overcome for their intensive application as tissue engineering scaffolds.

Various synthetic polymers have been utilized as blood vessels demonstrating good biomechanical properties, including poly-*ε*-caprolactone (PCL), poly-lactic acid (PLA), polyurethane (PU), and poly (lactide-*co*-*ε*-caprolactone) (PLCL). The mechanical properties as well as the cell response of fibrous scaffolds of these polymers vary based on the elasticity of used polymer, the thickness of the fibers, and the treatment employed before and after fabrication, including sterilization of α- or γ-radiations. Bilayered scaffolds of PCL/PLA demonstrate enhanced mechanical properties in comparison to pure PCL (Young’s modulus: 30.9 ± 6.6 MPa versus 10.7 ± 0.3 MPa). The wall thickness of PLCL fibrous scaffold affects its compliance. The thinner wall thickness is, the more compliant is the graft. The incorporation of hirudin into PLA enhances the Young’s modulus from 3.5 to 11.1 MPa after six months of implantation as well as inhibits platelet aggregations. The patency rate of poly(ester urethane) urea (PEUU) experiences increment from 40% to 92% when being implanted in abdominal rate for 24 weeks due to the functionalization by nonthrombogenic 2-methacryloyloxyethyl phosphorylcholine (MPC) copolymer. Increasing the porosity of polyurethane fibrous scaffold from 53% to 80% leads to an increment of cell population upon implantation in rate model. Dynamic culture of SMCs in bioreactor using PLCL fibrous scaffold show better collagen and DNA expressions compared to static culture for two weeks. The incorporation of drug such as DPA into BPU fibrous scaffold enhances both the tensile strength from 3.4 ± 0.4 MPa to 7.4 ± 0.1 MPa and ECs proliferation, while it inhibits SMCs proliferation.

Silk fibrous scaffold as natural polymer has contributed to the fabrication of SDBVs. Collagen promoted the formation of vessel-like tissue better than gelatin when blended with PCL, which in turn increases the young’s modulus from 1.77 ± 0.09 MPa to 5.99 ± 0.80 MPa upon implantation in nude mice for six months. Gelatin fibrous scaffolds show enhanced mechanical properties as a result of crosslinking by 15 mL 25% glutaraldehyde (for treated gelatin 33.8 MPa versus 5–10 MPa for untreated gelatin). Nevertheless, the strain to failure is still low comparable to natural arteries (11.7% versus 35% for artery). Elastin improves strain to failure although it decreases the modulus.

Hybridization of polymers provides an advanced strategy for the combination of good mechanical properties and cell interaction with the scaffold materials. Several polymer blends whether synthetic polymer blends or synthetic-natural polymer blends or natural polymer blends have all been employed for the fabrication of SDBVs.

In addition to the previous attempts, the incorporation of antithrombogenic agents, such heparin and lecithin, can lead to enhancement in the overall properties, along with the suppression of platelet adhesion. PCl-heparin conjugate results in higher relative growth rate (RGR) (160%) than pure PCL (100%) when cultured by ECs. Moreover, PLLA/heparin conjugate show higher patency rate (85.7%) than that of pure PLLA (40%). Lecithin has/zwitterionic surface nature, which decreases hemolysis ratio (HR) from 2.8% to 0.5% when conjugated with Chol-PCL scaffolds. Up to this end, a match between synthetic blood vessels and native blood vessels in terms of composition and function may be reachable by appropriate hybridization of polymers.

## Figures and Tables

**Table 1:** Mechanical properties of natural blood vessels.

Types	Elastic Modulus (MPa)	Ultimate Stress (MPa)	Strain at Failure (%)	Burst Strength (mmHg)	Ref.
Saphenous vein (Circ.)	43	3	11	NA	[17]
Saphenous vein (Long.)	130	13	17	NA	[17]
Saphenous vein (Circ.)	4.2	1.8	242	1680–3900	[18]
Saphenous vein (Long.)	23.7	6.3	83	NA	[18]
Saphenous vein (Circ.)	2.25	4	180	1250	[19]
Left internal mammary artery (Circ.)	8	4.1	134	2000	[18]
Left internal mammary artery (Long.)	16.8	4.3	59	NA	[18]
Femoral artery (Circ.)	9–12	1–2	63–76	NA	[20,21]

Circ.: circumferential; Long.: longitudinal; NA: not available.

**Table 2:** Small diameter blood vessels prepared and their mechanical properties.

Polymers	Solvents	Operating Conditions						Mechanical Properties				Ref.
		Polymer Concentration (*w*/*v* %)	Voltage (kV)	Air Gap (cm)	Flow Rate (mL/h)	Spinning Time (min)	Mandrel Rotation Speed (rpm)	Young’s Modulus (MPa)	Maximum Stress (MPa)	Maximum Strain (%)	Burst Strength (mmHg)	
**Synthetic polymer-based scaffolds**												
PCL-PLA	CHCl_3_	12.5	13	20	0.6	180	3600	30.9 ± 6.6	4.3 ± 0.2	47.0 ± 6.3		[34]
	CHCl_3_/DMF	14	13	20	1.5	180	10800	10.7 ± 0.3	1.2 ± 0.1	260		
PCL	CHCl_3_/EtOH	15	20		12	6			4.8	600		[43]
PCL	CHCl_3_/EtOH	5–15	15–25		12–24		4500		2–7.4	200–1200		[31]
TIPS-PEUU	HFIP	8	10		1		250	1.4 ± 0.4	8.3 ± 1.7			[19]
PCL	CHCl_3_/EtOH	15	20		12	6			4.1 ± 0.5	1092 ± 28	3280 ± 280	[30]
PLCL	HFP	9	15		1		500	1.2 ± 0.3	3.23 ± 0.57	270	933 ± 22	[35]
PCL	CHCl/MeOH	5	18		2			17.44 ± 0.91	13.35 ± 1.47	168.4 ± 8.76		[44]
		5	11		8			21.00 ± 1.39	8.72 ± 0.84	639.2 ± 24.15		
**Natural polymer-based scaffolds**												
Silk			10–11		0.9		3000	2.45 ± 0.47	2.42 ± 0.48		811	[45]
Gelatine	TFE	10	30		1.5	50	2	33.8	2.9	11.7		[49]
(rTE)	HFP	15	18.5	12.5	2		4400	0.91 ± 0.16	0.36 ± 0.05		485 ± 25	[53]
**Hybrid polymer-based scaffolds**												
PDO-elastin (50:50)	HFP	100 g/mL and 200 mg/mL	22	12	4 and 8		500	9.64 ± 0.66	3.25 ± 0.24	64.93 ± 3.97		[59]
Collagen-elastin-PLGA	HFP	−20	22	10	3		500	0.85	0.37			[60]
PLLACL coated with collagen	DCM/DMF	0	10		1	5	150	16.6 ± 4.4	3.9 ± 0.3	292 ± 87		[54]
PEUU-PMBU	HFP	15	10	15	1	5	250	3 ± 1	342 ± 43			[63]
PLA-Silk Fibroin-Gelatin	formic solution	13	30	13	0.2		1000		2.21 ± 0.18	60.58 ± 1.23	1596 ± 20	[64]
	CHCl_3_/EtOH	5	25	15	0.1		2000					
PCL-collagen	HFP	1	20	10	3		1000	2.7 ± 1.2	4.0 ± 0.4	140 ± 13	4915 ± 155	[65]
PHBV-PCL	CHCl_3_	1	20	15	0.5		3000	22 ± 7	1.4 ± 0.3	30 ± 20		[66]
Collagen-hitosan-P(LLA-CL)	HFP/TFA		14	12–15	1			10.3 ± 1.1	16.9 ± 2.9	112 ± 11	>3365 ± 6	[67]
Lecithin-cholesterol-(Chol-PCL)	CHCl_3_/DMF		18	15	3			35.92 ± 4.75	5.22 ± 0.50	107.15 ± 10.78		[68]

HFP: 1,1,1,3,3,3-hexafluro-2-propanol; TFE: 2,2,2-trifluoroethanol; DCM: dichloromethane; DMF: *N*,*N*-dimethylformamide; TFA: 2,2,2-trifluoroacetic acid.

**Table 3:** Polymers used for the fabrication of small diameter blood vessels and their biostudies.

Polymers	Cell Response		Ref.
	In Vitro Study	In Vivo Study	
**Synthetic Polymer-based Scaffolds**			
PCL-PLA	3T3 mouse fibroblasts cells covered the surface of PCL/PAL fibrous scaffold after 4 weeks. Human venous myofibroblasts (HVS) cells were concentrated in the outer layer of PCL-PLA scaffold.		[34]
PCL		Implanted in a rat revealing that endothelilization and extra cellular matrix (ECM) formation of PCL was faster than PTFE commercial grafts.	[49]
PCL		In vivo implantation in rat for 12 weeks showed that the blood vessels were completely endothelilized with thrombosis formation.	[31]
TIPS-PEUU	Cell culture resulted in density up to 92 ± 1% using Adult stem cells.		[19]
PCL		Good patency rate, no thrombosis formation and rapid endothelilization up to 6 months of implantation in abdominal rat aorta. However, calcium deposition appeared after that at longer term of implantation.	[30]
PLCL	Smooth muscle cells (SMCs) were cultured for up to 7 weeks. The viability of cells increased by increasing cell culture time (11 × 10^5^ cells after 7 weeks).		[35]
PCL	Thicker fiber diameter based PCL graft enhanced the formation of immunomodulatory and tissue remodeling (M2) phenotype when MSCs cells were cultured.		[50]
**Natural Polymer-based Scaffolds**			
Silk	Human aortic endothelial cells and coronary artery smooth muscle cells experienced good proliferation.		[51]
(rTE)	Tropoelastin based blood vessel showed good endothelial cell response in terms of adhesion and proliferation.		[49]
**Hybrid Polymer-based Scaffolds**			
PDO-Elastin (50:50)	Human dermal fibroblasts cells cultured on pure PDO and PDO-elastin blend for 7 days. Hybrid scaffold of PDO-elastin showed better cell response than pure PDO in terms of adhesion, proliferation and migration.		[59]
Collagen-elastin-PLGA	Ovine SMCs cultured on collagen/elastin/PLGA blend for 7 days demonstrating good cell viability (90%).		[60]
PLLACL coated with collagen	P LLA-CL-collagen vascular graft demonstrated good cell response when HCAECs are cultured.	P(LLA-CL)/collagen vascular graft demonstrated good patency without thrombosis formation when implanted in rabbit veins.	[54]
PEUU-PMBU	Rat smooth muscle cells were cultured on PEUU/PMBU fibrous scaffold for 1 day resulting in diminishment of cell number (70–76%) compared to the control (TCPS) and pure PEUU.	Implanting the PEUU/ PMBU fibrous scaffold in rat abdominal aorta showed higher patency than PEUU.	[63]
PLA-silk Fibroin-Gelatin	3T3 mouse fibroblast cells cultured for 21 days on PLA/SF-gelatin showed good proliferation.	Subcutaneous implantation test in Sprague-dawley rat for 3 months resulted in biocompatibility of the graft.	[64]
PCl-Collagen	Bovine endothelial cells (bECs) and smooth muscle cells (SMCs) were cultured on PCL-collagen fibrous scaffold demonstrating confluent layer of ECs on the lumen of the graft.		[65]
PHBV-PCL	RCEC cells experienced apoptosis on PHBV because of its stiffness.		[66]
Collagen-Chitosan-P(LLA-CL)	ECs cells demonstrated good adhesion and proliferation on collagen-chitosan-P(LLA-CL) compared to pure P(LLA-CL).		[67]
Lecithin-cholesterol-PCL	MSC cells were cultured for 7 days on both pure Chol-PCL and lecithin-Chol-PCL for 7 days. MSCs proliferated better on lecithin doped Chol-PCL.		[68]
